# Supplementary material for: Research on local sound field intensity control technique in metasurface based on deep neural networks
Source: PLoS One. 2024 Mar 28;19(3):e0301211. doi: 10.1371/journal.pone.0301211 (PMC10977772; doi:10.1371/journal.pone.0301211)
Supplement: Attachment — Submitted filename: review.docx [file pone.0301211.s001.docx]

This paper introduces a novel metasurface inverse design method leveraging parallel deep neural networks (PDNN). The method respectively extracts the key in formation of the acoustic field and the metasurface as the input and output of the PDNN network. With the help of the kernel loss function and the constraint performance provided by the constraint network, it establishes an inverse mapping relationship between the target acoustic field and the physical structure parameters of the metasurface. The method has certain design potential and may find applications in the field of underwater acoustic stealth. However, before accepting the publication of this paper, it is necessary to address the following questions.

1. The state-of-the-art review in the introduction section is a bit poor. In particular, the description of metasurfaces and pentamode metamaterials is a bit too fast citing only a few papers for each mentioned field and does not give proper credit to previous literature contributions. The manipulation of scattering characteristics in the paper aligns with the concept of coding metasurfaces [Natl. Sci. Rev. 2022, 9, nwac030; Phys. Rev. Appl. 2022, 17, 034019; Small 2024, 2308349, DOI: 10.1002/smll.202308349]. The conceptual framework for the design of the pentamode metamaterial also requires richer literature support [J Acoust Soc Am 2009; 125: 839-849; J Sound Vib 2019; 443: 238–252; J Mech Phys Solids 2021; 152:104407].
2. This paper is limited to the optimization design of equivalent parameters and does not delve into more complex pentamode metamaterial configuration designs. To enhance the completeness of the paper, it is suggested to provide potential structural instances based on the designed parameter distribution. Even referencing cases from the literature would be beneficial in demonstrating the effectiveness of the design and the rationality of the designable parameters.
3. Since this paper does not involve the design of actual configurations and lacks experimental validation, the use of the term "experiment" in the main text is inappropriate.
4. Does the scattering wave control strategy based on machine learning offer the advantage of faster and more convenient implementation? If so, does this attribute hold potential for applications in three-dimensional metasurface design and even curved metasurface design? Please provide further insights on this matter.
5. This manuscript primarily focuses on the equivalent parameter design of underwater low-detectability metasurfaces. It is recommended to enhance its functionality, for instance, by comparing the reflection energy of this scattering metasurface with that of a fully reflective metasurface to evaluate the metasurface's acoustic target characteristics.
